# Monitoring individualized glucose levels predicts risk for bradycardia in type 2 diabetes patients with chronic kidney disease: a pilot study

**DOI:** 10.1038/s41598-024-81983-x

**Published:** 2024-12-05

**Authors:** Pejman Farhadi Ghalati, Moein E. Samadi, Marlo Verket, Paul Balfanz, Dirk Müller-Wieland, Stephan Jonas, Andreas Napp, Christoph Wanner, Markus Ketteler, Athina Vassiliadou, Stefan Heidenreich, Thomas Deserno, Gudrun Hetzel, Danilo Fliser, Malte Kelm, Jürgen Floege, Nikolaus Marx, Andreas Schuppert

**Affiliations:** 1 Institute for Computational Biomedicine, RWTH Aachen University, Aachen, Germany; 2 Department of Internal Medicine I, University Hospital RWTH, Aachen, Germany; 3 Institute for Digital Medicine, University Clinic Bonn, Bonn, Germany; 4 Department of Internal Medicine I, University Hospital Würzburg, Würzburg, Germany; 5 Department of Nephrology, Coburg, Germany; 6 Department of Nephrology and Diabetology, Kliniken Maria-Hilf GmbH, Mönchengladbach, Germany; 7 KFH Nephrology Center, Aachen, Germany; 8 Peter L. Reichertz Institute for Medical Informatics of TU Braunschweig and Hannover Medical School, Braunschweig, Germany; 9 Department of Nephrology, Düsseldorf, Germany; 10 Department of Internal Medicine IV, Saarland University Medical Centre, Homburg, Germany; 11 Department of Cardiology, Pulmonology and Vascular Medicine, HHU Düsseldorf, Düsseldorf, Germany; 12 Department of Internal Medicine II, University Hospital Aachen, RWTH Aachen University, Aachen, Germany

**Keywords:** Chronic kidney disease, Bradycardia, Diabetes mellitus, Hypoglycemia, Glucose monitoring, Personalized medicine, Machine learning, Chronic kidney disease, Arrhythmias, Risk factors, Endocrine system and metabolic diseases, Machine learning, Data processing

## Abstract

Patients with diabetes mellitus (DM) and chronic kidney disease (CKD) exhibit an elevated risk for cardiac arrhythmias, such as bradycardia, which may potentially lead to sudden cardiac death (SCD). While hypoglycemia, defined as a critical drop in glucose levels below the normal range, has long been associated with adverse cardiovascular events, recent studies have highlighted the need for a comprehensive reevaluation of its direct impact on cardiovascular outcomes, particularly in high-risk populations such as those with DM and CKD. In this study, we investigated the association between glucose levels and bradycardia by simultaneously monitoring interstitial glucose (IG) and ECG for 7 days in insulin-treated patients with DM and CKD. We identified bradycardia episodes in 19 of 85 patients (22%) and associated these episodes with personalized low, medium, and high relative glucose levels. Our analysis revealed a significant increase in bradycardia frequency during periods of lowest relative glucose, particularly between 06:00-09:00 and 12:00-15:00. Furthermore, leveraging a Random Forests classifier, we achieved a promising area under the curve (AUC) of 0.94 for predicting bradyarrhythmias using glucose levels and heart rate variability features. Contrary to previous findings, only 4% of bradycardia episodes in our study population occurred at glucose levels of 70 mg/dL or lower, with 28% observed at levels exceeding 180 mg/dL. Our findings not only highlight the strong correlation between relative glucose levels, heart rate parameters, and bradycardia onset but also emphasize the need for a more personalized definition of hypoglycemia to understand its relationship with bradyarrhythmias in high-risk DM and CKD patient populations.

## Introduction

Patients with diabetes mellitus (DM), particularly those with a prolonged history of diabetes, insulin therapy, and chronic kidney disease (CKD), are considered vulnerable individuals with an elevated risk of experiencing cardiac arrhythmias and sudden cardiac death (SCD)^1–3^. Various factors contribute to the development of potentially fatal arrhythmias in patients, including coronary heart disease^4^, diabetes- and CKD-induced cardiomyopathy^5,6^, and autonomic neuropathy^7^. Among these, hypoglycemic events are regarded as direct triggers for such arrhythmias^8–10^. Hypoglycemia arises when glucose levels drop below the normal range to a critical level. In individuals with DM, hypoglycemia can occur as an adverse complication of insulin treatment^11,12^.

In 1991, Tattersall and colleagues were the first to describe the phenomenon of sudden nocturnal death in young patients with T1DM^13^. They reported that many of these patients had experienced recent episodes of nocturnal hypoglycemia. Consequently, it has been suggested that severe hypoglycemia might trigger cardiac arrhythmias, a concept later coined as the “dead in bed” syndrome^14^. Additionally, findings from extensive cardiovascular outcome trials in patients with T2DM suggest that severe hypoglycemia is associated with an increased risk of cardiovascular events and cardiovascular-related mortality^15^. Moreover, CKD substantially raises the susceptibility to hypoglycemia, with even moderate impairment of kidney function, estimated glomerular filtration rate (eGFR) < 60 ml/min, associated with a noteworthy increase in SCD^16^.

Clinical and empirical evidence indicate that hypoglycemia can induce fatal arrhythmias by causing alterations in cardiac repolarization in individuals with diabetes^17–19^. Arrhythmia refers to irregular heartbeat patterns and is a significant cardiovascular risk factor. Various studies suggest a robust association between hypoglycemic events and cardiovascular mortality^20^, underscoring their clinical importance. In particular, Chow et al.^8^ demonstrated a notable rise in the occurrence of bradyarrhythmias among T2DM individuals, while Novodvorsky et al.^21^ observed a similar trend in T1DM patients. Bradycardia, characterized by heart rates falling below a defined threshold, represents one of the prevalent types of cardiac arrhythmias.

However, recent findings from our group revealed bidirectional associations between hypoglycemia and cardiovascular outcomes in the CARMELINA trial. Surprisingly, in the CAROLINA trial, no associations were observed in either direction, questioning the conventional belief that hypoglycemia directly leads to adverse cardiovascular events^22^. To address this open question, our study explored the correlation between hypoglycemic glucose levels and bradycardia using simultaneous 12-lead Holter-ECG and continuous glucose monitoring data over 7 days in insulin-treated individuals with diabetes and CKD, a population with a high susceptibility to hypoglycemic events.

Several studies have employed machine learning models to predict bradyarrhythmia. By utilizing preoperative and real-time intraoperative data, researchers have developed predictive models to detect unstable bradycardia during surgical procedures. These machine learning models have demonstrated positive predictive values with 95% specificity and Area Under the Curve (AUC) scores ranging from 0.81 to 0.89^23^. Another study^24^ demonstrated the utility of heart rate variability (HRV) features in predicting the risk of bradycardia in patients experiencing chest pain. Hanns et al.^25^ revealed that preoperative HRV in high-risk patients could indicate hemodynamic events with both high sensitivity and specificity. Furthermore, researchers have suggested the application of artificial neural networks to predict the onset of bradycardia in preterm infants through the continuous monitoring of vital signs^26^. These studies indicate that HRV and various vital sign parameters can serve as predictive indicators for bradycardia across diverse clinical settings. Machine learning methods can be employed to construct practical models for risk stratification and decision support based on these indicators. Notably, despite these advancements, no study has explored the potential of incorporating temporal glucose measurements and heart rate parameters to predict bradycardia in patients with diabetes.

In this study, we employed a multi-step approach to investigate the relationship between glucose levels and bradycardia events in patients with diabetes and CKD. First, we identified patients who developed bradycardia within the overall cohort and compared their baseline characteristics to those without bradycardia. Second, utilizing a personalized definition for extreme glucose, we established the statistical correlation between bradycardia events and personalized *relative glucose levels* by analyzing the association within the bradycardia group and treating each patient as their own control to account for confounding variables. Third, we examined the relationship between HRV features and bradycardia events and constructed a predictive model by employing feature engineering on heart rate, glucose, and HRV features. We utilized a Random Forest classifier to forecast bradycardia occurrences using these features, which was achieved through our custom-developed Python software designed for time series data preprocessing and feature engineering.

## Material and methods

### Data resources

*Patients.* We analyzed data from two initial cohorts with a total of 124 insulin-treated patients with diabetes and an eGFR < 40 ml/min/1.73m2 (predialysis cohort; n=62; NCT02315300) or on maintenance dialysis treatment for at least 3 months (dialysis cohort; n=62; recruited in 6 kidney centers; NCT02001480). The predialysis cohort recruited patients with T1DM or T2DM while the dialysis cohort enrolled only patients with T2DM. Exclusion criteria were as follows: Pregnancy or women without sufficient contraception, adapted specifically to amenorrheic hemodialysis patients; life expectancy below 6 months; participation in another clinical trial within the previous 2 months; history of any other illness, any current or past medical condition and/or required medication to treat a condition that could affect the evaluation of the study; alcohol or drug abuse; patient committed to an institution by legal or regulatory order; expected non-compliance; patients unwilling or unable to give informed consent, or with limited ability to comply with instructions for this study.

*Monitoring.* All patients underwent 7-day simultaneous 12-lead Holter-ECG as well as continuous IG monitoring while continuing their daily activities and diabetes management. 12-lead ECG (12-lead ECG system medilog® DARWIN FD12 from Schillermed) recordings were performed at a 1000-Hz sampling rate with electrodes using the Mason-Likar configuration. Timesynchronized CGM was performed with IG measurement every minute and 5 min averages were reported employing the CGM system G4 from Dexcom. Calibrations were performed at least 3 times during the 7 day period. Data handling was supported by web services integrating OpenClinica with binary large objects (BLOBs) processing.

Arrhythmia analysis was performed using the proprietary Software Darwin 2 (Schillermed). Bradycardia is defined as having more than 4 consecutive beats at a heart rate of fewer than 45 beats per minute. Arrhythmia and ECG signal data was exported to MATLAB (the MathWorks) and time-aligned with CGM data for further statistical analysis.

*Data preparation.* We performed analyses of valid simultaneous ECG and continuous glucose recordings over 7 days in 85 of the 124 patients enrolled. Patients were excluded from analysis because of the following reasons: (1) missing or incomplete ECG data (e.g., disconnection of ECG N=18); (2) missing glucose data (e.g., device error N=11); (3) violating of inclusion criteria without prior indication (i.e., pacemaker rhythm visible in ECG N=8); (4) missing time synchronization of ECG and glucose data (N=1); (5) study drop-out (N=1). The data analyzed included three main recordings of 85 patients measured over 7 days: RR intervals, interstitial glucose levels, and bradycardia events. The RR intervals were extracted from simultaneous 12-lead Holter-ECG records with a sampling rate of 1000 Hz, using the proprietary software Darwin 2 (Schillermed). The extracted RR intervals were used later for arrhythmia analysis to detect bradycardia episodes. The interstitial glucose was recorded every minute, and 5-minute averages were reported. It is measured using a sensor filament usually placed on the abdomen and not close to the insulin pump injection side. It should be noted that during the monitoring, subjects continued their daily activities and diabetes management, while sensor calibrations were performed at least 3 times during the 7 days.

### HRV analysis

Heart rate is the reciprocated value of the distance between two consecutive beats (R peaks in an Electrocardiogram (ECG) signal). A healthy heart is not a constant value and shows complex and nonlinear fluctuations, while cardiac abnormalities increase or decrease such complex oscillations^27^.

HRV assesses changes in the RR intervals. In other words, HRV quantifies spatial and temporal complexity through time and frequency heart rate parameters^28^. Significant changes in resting HRV parameters can be associated with cardiac diseases or emotional conditions; therefore, HRV features provide a powerful tool for identifying and characterizing the cardiac health status of patients^29^.

HRV can be defined using time-domain, frequency-domain and nonlinear features in different time intervals: 24 hours, short-term ($\sim$ 5 minutes), and ultrashort-term (< 5 minutes)^29^. As the glucose sampling rate is 5 minutes, we used short-term HRV analysis in the present study.

*HRV time domain features.* Time-domain features of HRV estimate the RR intervals’ variability in a monitoring span (here 5 minutes). We used the RR intervals time-domain features used in this study, suggested by Shaffer and Ginsberg^29^. We also defined instantaneous heart rate time-domain parameters. These features are depicted in Table 1.

**Table 1:** Description of HRV time-domain features.

**Feature**	**Unit**	**Description**
RR-sdnn	ms	Standard deviation of RR intervals
RR-pnn50	%	Percentage of consecutive RR intervals that vary by more than 50 ms
RR-pnn20	%	Percentage of consecutive RR intervals that vary by more than 20 ms
RR-rmssd	ms	Root mean square of consecutive RR interval differences
HR-mean	bpm	The mean value of heart rates
HR-median	bpm	The median value of heart rates
HR-std	bpm	The standard deviation value of heart rates
HR-max	bpm	The highest value of heart rates
HR-min	bpm	The lowest value of heart rates
HR-p2p	bpm	(peak to peak) The difference of the highest and lowest values of heart rates
HR-kurtosis	-	The kurtosis value of heart rates
HR-skewness	-	The skewness value of heart rates
HR-mad	bpm	The median value of absolute changes of heart rates
HR-zcr	-	The normalized zero-crossing rate of heart rates

*HRV spectral domain features.* Frequency-domain features quantify the HRV in spectral space by using the Fourier transform to estimate absolute or relative power in specific frequency bands. The Task Force of the European Society of Cardiology and the North American Society of Pacing and Electrophysiology (1996)^29^ suggested four frequency bands representing HR fluctuations in the frequency domain: The ultra-low-frequency (ULF) band ($\le$0.003 Hz), the very low frequency (VLF) band (0.0033-0.04 Hz), the low frequency (LF) band (0.04-0.15 Hz), and the high frequency (HF) or respiratory band (0.15-0.40 Hz). Since ULF requires a recording interval of at least 24h^30^, we did not use this parameter in this research. Table 2 shows the parameters defined in VLF, LF, and HF bands.

**Table 2:** Description of HRV frequency-domain features.

**Feature**	**Unit**	**Description**
RR-vlf-power	ms^2^	Absolute power of the very-low-frequency band (0.0033-0.04 Hz)
RR-lf-power	ms^2^	Absolute power of the low-frequency band (0.04-0.15 Hz)
RR-hf-power	ms^2^	Absolute power of the high-frequency band (0.15-0.4 Hz)
RR-total-power	ms^2^	Absolute power of the very-low to a high-frequency band (0.0033-0.4 Hz)
RR-lf-norm	%	Relative LF power to the sum of LF and HF power
RR-hf-norm	%	Relative HF power to the sum of LF and HF power
RR-lf-peak	Hz	Peak frequency of the low-frequency band (0.04-0.15 Hz)
RR-hf-peak	Hz	Peak frequency of the high-frequency band (0.15-0.4 Hz)
RR-lfhf-ratio	%	Ratio of LF-to-HF power

Performing Fourier transform on a time series requires an invariant sampling rate, while the RR intervals data by nature is unequally spaced sampled. Hence, before HRV frequency-domain extraction, the data is interpolated using a cubic polynomial on a 4 Hz constant sampling frequency time span.

*HRV nonlinear features.* RR interval measurements exhibit highly complex and irregular dynamics. We can use nonlinear parameters to assess the randomness and nonlinearity of a heart rate time series. Table 3 shows the employed nonlinear features using entropy analysis and the Poincaré plot.

Poincaré plot is a type of recurrence plot used to estimate self-similarity in a system’s dynamic. For HRV analysis, the Poincaré plot is a scatter plot of RR intervals against the previous interval ($RR_t$ vs. $RR_{t-1}$). A Poincaré plot can be explored by fitting an ellipse to the plotted points, and the corresponding area and diameters of the ellipse can be extracted as nonlinear features. The area represents total variability, the length of the fitted ellipse measures short-term and long-term variability, while the width quantifies the short-term HRV^30^.

For the entropy analysis, we used sample entropy of 2-dimensional embedded space and permutation entropy of 3-dimensional embedded space with one-step lag. Sample entropy, a derivation of Shannon entropy, is a measure for estimating the complexity of dynamic physiological systems based on the existence of patterns^31^. Permutation entropy is another derivation of Shannon entropy, used to assess the complexity of time series data, proposed by Bandt^32^. It quantifies the complexity of a system based on similar ordinal patterns.

**Table 3:** Description of HRV nonlinear features.

**Feature**	**Unit**	**Description**
RR-sd1	ms	Width of the fitted ellipse on the Poincaré plot
RR-sd2	ms	Length of the fitted ellipse on the Poincaré plot
RR-s-area	ms	Area of the fitted ellipse on the Poincaré plot
RR-sd-ratio	-	Ratio of *SD*1-to-*SD*2
RR-sample-ent	-	Sample entropy, measures the irregularity and randomness of a time series
RR-perm-ent	-	Permutation entropy, measures the irregularity and randomness of a time series

### Data preprocessing and feature extraction

*Windowing.* As mentioned in the HRV analysis section, due to the 5-minute sampling rate of glucose measurement in the data, we performed a short-term (5-minute) HRV analysis. Therefore, the feature extraction process was performed in a moving window fashion. The length of the window was set to 5 minutes with a similar step size, resulting in no overlaps between the windows. To lower the impact of high variance in the number of bradycardias, we considered all bradyarrhythmias in a 5-minute window as one Brady episode. We extracted HRV features and the measured glucose value within each window. Additionally, to study the daytime effect, we calculated a continuous form of the 24-hour clock by decomposing the time into a Sin and Cosine function with 24-hour periodicity, hereinafter referred to as C24-sin and C24-cos, respectively.

The required denoising and missing value imputation prior to feature calculation were conducted in the workflow of windowing the RR intervals and glucose values, as explained in the Supplementary Methods.

### Statistical analysis

Continuous variables were compared between groups using the Mann-Whitney U test. This test was applied to assess differences in demographic and clinical characteristics between patients with and without bradycardia, as well as to compare the frequency of bradycardia events across relative glucose levels. Categorical variables were analyzed using Fisher’s exact test, including the demographic and clinical characteristics of patients with and without bradycardia. Additionally, Fisher’s exact test was used to analyze bradycardia episodes at different glucose levels at different time points during the day. Fisher’s exact test, introduced by Ronald Fisher^33^, is a statistical significance test based on the hypergeometric distribution and examines the relationship between two categorical variables using contingency table analysis. Wilcoxon Ransum significance test was also utilized to analyze the association between HRV features and bradycardia events.

### Implementation

A time-series analysis Python package for continuous monitoring data analysis (CMDA)^34^, was utilized to perform all the necessary pre-processing steps, encompassing windowing, filtering, and missing value imputation. The subsequent statistical and machine learning analyses were carried out in Python, employing the Scipy^35^ and Scikit-learn^36^ packages.

### Ethical approval

All methods were performed in accordance with the local Ethics Committee NCT02315300 - EK096/16 and NCT02001480 - EK187/12 guidelines and regulations. All patients involved in this study gave informed consent and all experimental protocols were approved by the local Ethics Committee (EK096/16 and EK187/12).

## Results

### Patient characteristics

Bradycardia, defined as > 4 consecutive beats at RR < 1333 ms (instantaneous heart rate < 45 beats/min), occurred in 19 of 85 patients (22%); the number of episodes varied between 1 and 538 episodes with a mean of 106.4±157.6 episodes over 7 days. To reduce the effect of high variance in the number of bradycardias, we grouped dense clusters of bradycardias defining two arrhythmic episodes in one group when the time distance between them is less than 5 minutes. Table 4 presents the demographic and clinical characteristics of patients with and without episodes of bradycardia. Continuous variables were compared using the Mann-Whitney U test, while categorical variables were analyzed using Fisher’s exact test. Significant differences were observed in variables such as eGFR, mean heart rate, prior stroke, prior myocardial infarction, atrial fibrillation, and anticoagulation, with *P* values below 0.05 indicating statistical significance.

**Table 4:** Demographic data of patients with and without episodes of bradycardia in the study cohort

Baseline Variables	All patients	Patients with bradycardia	Patients without bradycardia	*P* value
Participants (n); male (%)	85; 69	19; 74	66; 68	0.63
Age (y)	$67.4 \pm 12.3$	$71.5 \pm 12.6$	$66.2 \pm 12.1$	0.18
Diabetes duration (y)	$19.6 \pm 13.2$	$17.7 \pm 12.5$	$20.2 \pm 13.5$	0.56
T1DM (%); count (n)	7.1; 6	5.3; 1	7.6; 5	0.72
Mean glucose [mg/dL]	$165.3 \pm 40.5$	$161.1 \pm 26.2$	$166.5 \pm 43.9$	0.67
Mean glucose in low relative glucose tertile [mg/dL]	$109.2 \pm 30.3$	$107.0 \pm 25.6$	$109.8 \pm 31.3$	<0.05
Mean glucose in middle relative glucose tertile [mg/dL]	$156.6 \pm 37.7$	$154.7 \pm 29.5$	$157.0 \pm 39.4$	<0.05
Mean glucose in high relative glucose tertile [mg/dL]	$220.8 \pm 59.7$	$220.7 \pm 48.6$	$220.9 \pm 52.1$	0.29
Mean eGFR (non-dialysis patients)	$27.1 \pm 16.8$	$37.8 \pm 20.8$	$25.1 \pm 15.7$	<0.05
Mean systolic BP (mmHg)	$138.9 \pm 23.2$	$145.8 \pm 22.2$	$136.9 \pm 23.3$	0.22
Mean diastolic BP (mmHg)	$71.6 \pm 13.4$	$69.5 \pm 16.3$	$72.2 \pm 12.5$	0.54
Mean Heart Rate [bpm]	$73.4 \pm 11.3$	$65.9 \pm 10.4$	$75.5 \pm 10.7$	<0.05
BMI (kg/$\hbox {m}^2$)	$31.7 \pm 7.7$	$32.9 \pm 8.6$	$31.3 \pm 7.4$	0.54
Smoking (%); count (n)	15.5; 13	21.1; 4	13.8; 9	0.46
Hypertension (%); count (n)	84.5; 72	89.5; 17	83.1; 55	0.52
Prior stroke (%); count (n)	16.5; 14	31.6; 6	12.1; 8	<0.05
Prior myocardial infarction (%); count (n)	35.3; 30	57.9; 11	28.8; 19	<0.05
Atrial fibrillation (%); count (n)	28.2; 24	52.6; 10	21.2; 14	<0.05
Coronary artery disease (%); count (n)	57.7; 49	73.7; 14	53.0; 35	0.14
Beta-blocker (%); count (n)	71.8; 61	68.4; 13	72.8; 48	0.76
Antiarrhythmic drugs (%); count (n)	5.9; 5	10.5; 2	4.5; 3	0.42
Antiplatelet therapy (%); count (n)	63.5; 54	63.2; 12	63.6; 42	0.98
Anticoagulation (%); count (n)	29.4; 25	52.6; 10	22.7; 15	<0.05
ACE inhibitors (%); count (n)	49.4; 42	42.1; 8	51.5; 34	0.53
Lipid lowering drugs (%); count (n)	65.9; 56	63.2; 12	66.7; 44	0.81
Diuretics (%); count (n)	76.5; 65	73.7; 14	77.2; 51	0.78

**Fig. 1:**
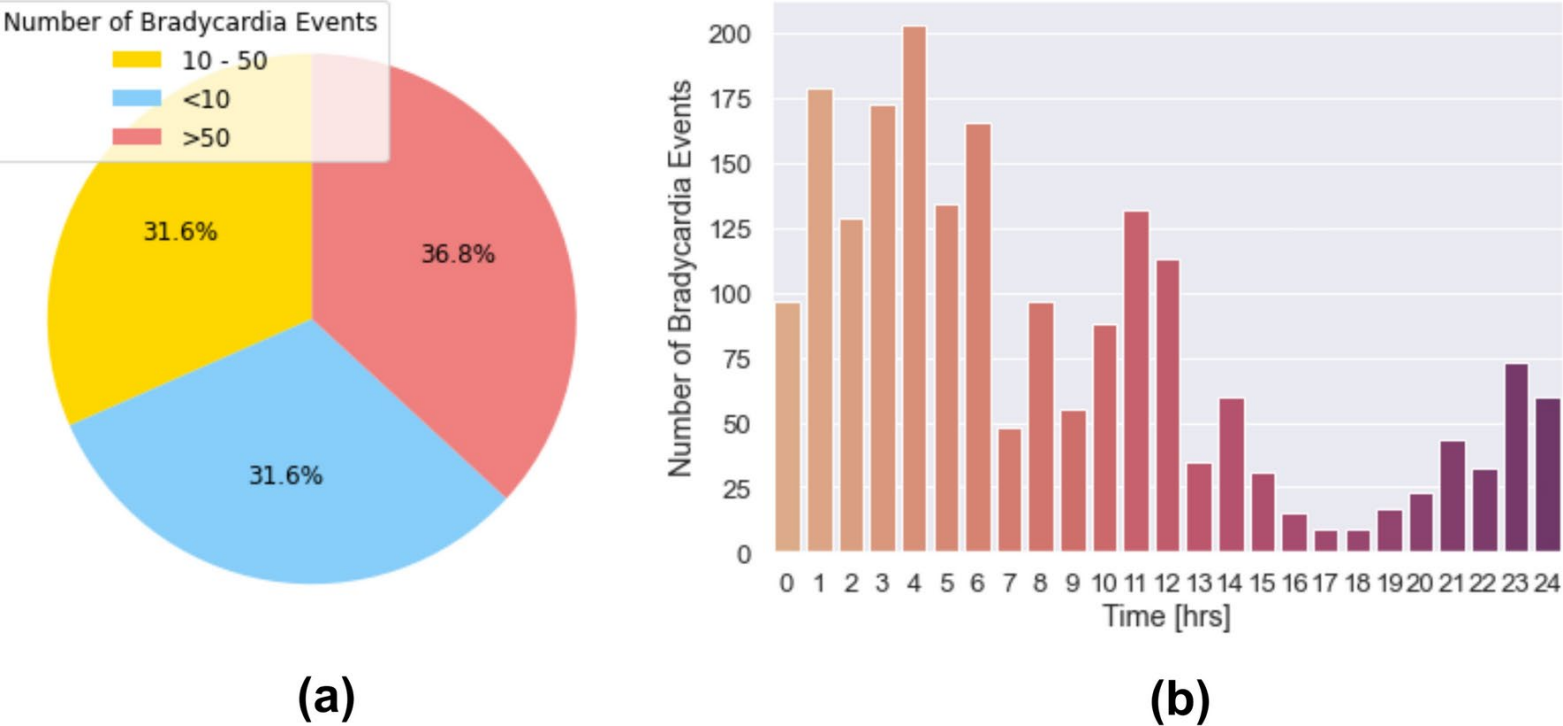
Distribution and number of bradycardia episodes. **(a)** Distribution of bradycardia episodes per patient, based on three frequency levels: less than 10 episodes, from 10 to 50 episodes, and more than 50 episodes. **(b)** Number of bradycardia episodes in all patients at every hour of the day.

Figure 1a illustrates the distribution of bradycardia event frequencies among the 19 patients with bradycardia. Notably, in more than a third of the patients, there are more than 50 bradyarrhythmias recorded over a 7-day period, while in another third, this frequency is less than 10. The occurrence time of bradycardias is another critical factor to be considered. Figure 1b shows an apparent increase in such events after midnight and before morning. The same pattern can be seen shortly before noon, while the number of bradycardias drops in the afternoon and evening. We studied this trend in detail in the next section.

### Association between glucose and bradycardia events

Given the hypothesis that extreme glucose levels may induce severe bradycardia in patients with diabetes, we analyzed the connection between the number of bradycardic events and the absolute glucose value. In all patients with bradycardias, the majority of bradycardias occurred at normal glucose levels above 70 mg/dL and below 180 mg/dL (72%), while only 4% of all bradycardia episodes arose at glucose values below 70 mg/dL.

**Fig. 2:**
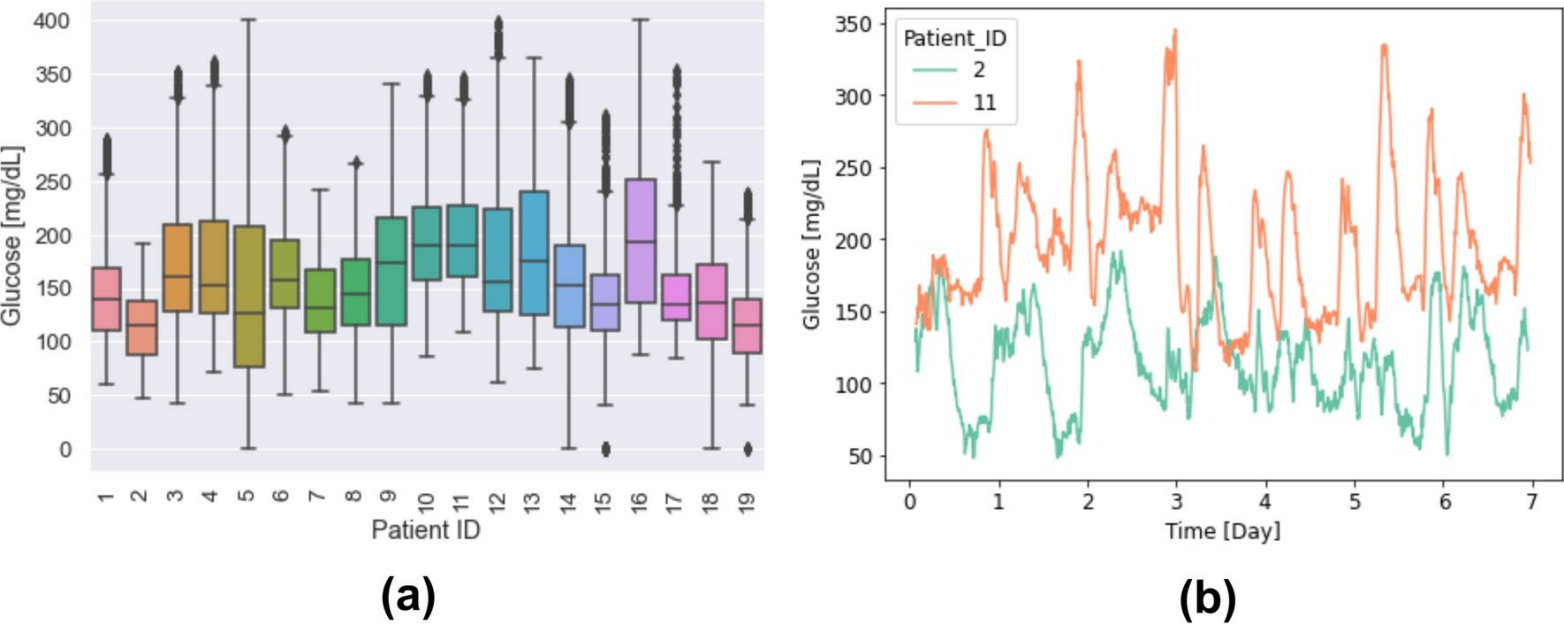
Absolute glucose range and example profiles. **(a)** Absolute glucose range in each of the 19 patients with bradycardia episodes. **(b)** Example of glucose profile of two patients with bradycardia episodes, which shows a clear difference in glucose level range in these two patients.

Further examinations of glucose profiles in the patients with bradycardia episodes indicated a high variability of glucose values over 7 days with significantly different mean glucose values and very dissimilar glucose profiles (Figure 2a). For illustration, Figure 2b displays the glucose profiles of two patients. Patient 2 has a mean glucose value of 197.2 mg/dL over 7 days, and despite its high glucose volatility, it never goes below 70 mg/dl. On the other hand, patient 11 underwent several hypoglycemia episodes with glucose measurements < 70 mg/dl.

Based on these results, we hypothesized that relative glucose values might be more effective than absolute glucose values for analyzing bradycardia in relation to glucose levels in these patients. Consequently, we categorized glucose levels into personalized tertiles for each patient. Specifically, we defined a personalized relative glucose level by observing the range of glucose variation for each patient over a day and dividing this range into three equal tertiles: the lower 33% labeled as low relative glucose level, the middle 33% as medium, and the upper 33% as high relative glucose level.

**Fig. 3:**
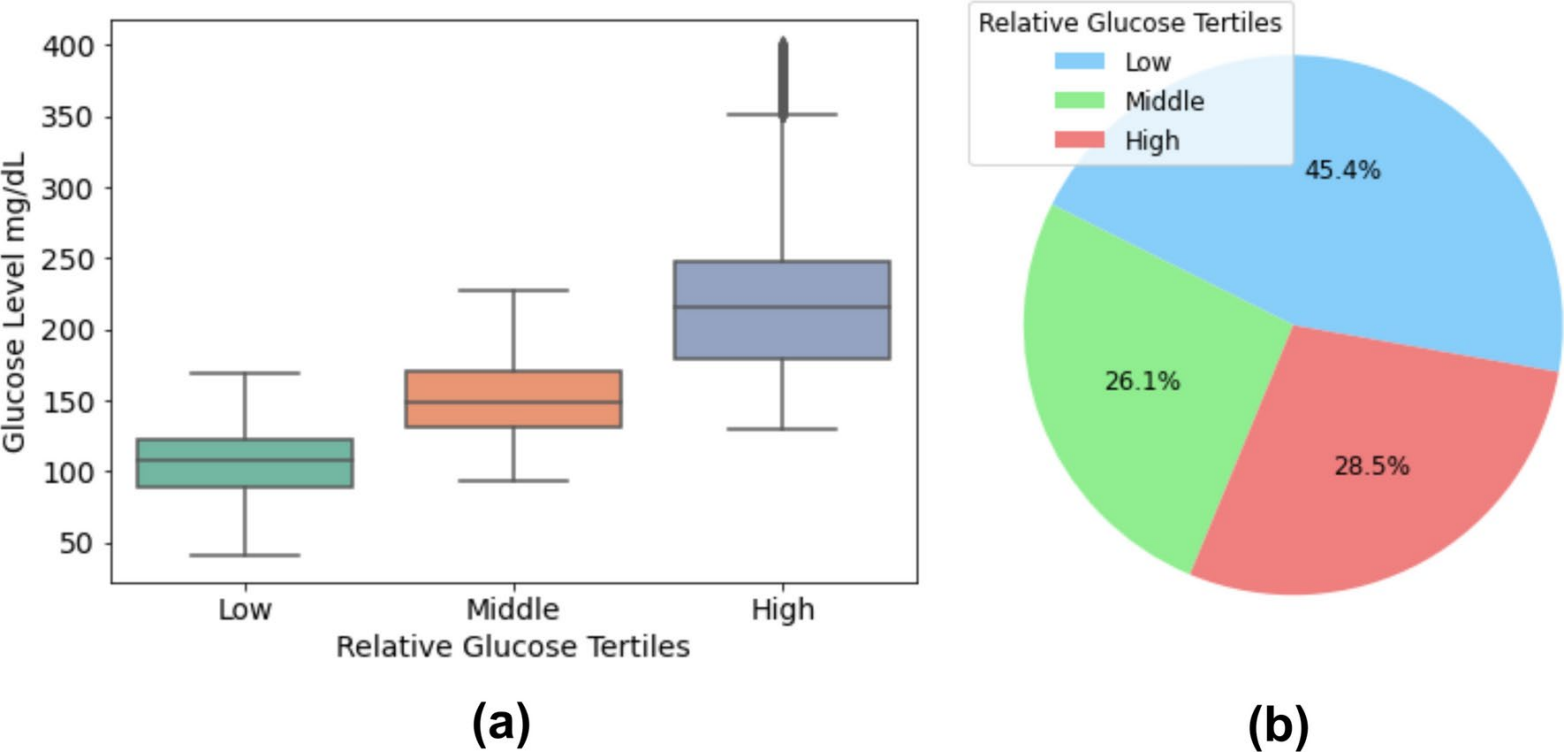
Relative glucose levels distribution and percentage. (**a**) Distribution of glucose levels in each relative individualized glucose tertile. **(b)** Percentage of bradycardia events at different glucose levels.

As expected, due to the different glucose profiles in the patients, we found an overlap in the glucose level distribution at each relative glucose tertile (Figure 3a). The relative categorization of glucose levels shows that more than 45% of bradycardias occurred in the low glucose level tertile, while around 28% of these events appeared at the high glucose level tertile (Figure 3b).

To evaluate the statistical significance of the observed unequal distribution of bradycardia events across relative glucose tertiles, we used the Mann-Whitney U test. This non-parametric test was chosen due to the non-normal distribution of bradycardia event frequencies and the varying sample sizes across the tertiles. The null hypothesis for the Mann-Whitney U test asserts that there is no statistically significant difference in the distribution of bradycardia events between the glucose tertiles under comparison.

Table 5 presents the results of the Mann-Whitney U test for pairwise comparisons of bradycardia event frequencies across the three relative glucose tertiles: low, middle, and high tertiles. At a significance level of $\alpha = 0.05$, the analysis revealed a statistically significant difference between the low and middle tertiles (U = 1376.0, *P* value = 0.015), allowing us to reject the null hypothesis. s Additionally, further analysis in the supplementary materials, examining the occurrence of bradycardia events across relative glucose tertiles, shows a significantly higher frequency of bradycardia events in the low glucose tertile. These findings suggest that glucose levels may influence the frequency of bradycardia events, particularly between the low and medium tertiles.

**Table 5:** Results of the Mann-Whitney U test for pairwise comparisons of bradycardia event frequencies across relative glucose tertiles.

Comparison between relative glucose tertiles	U-statistic	*P* value
Low tertile vs Middle tertile	1376.0	0.015
Low tertile vs High tertile	1269.0	0.051
Middle tertile vs High tertile	1115.0	0.520

**Fig. 4:**
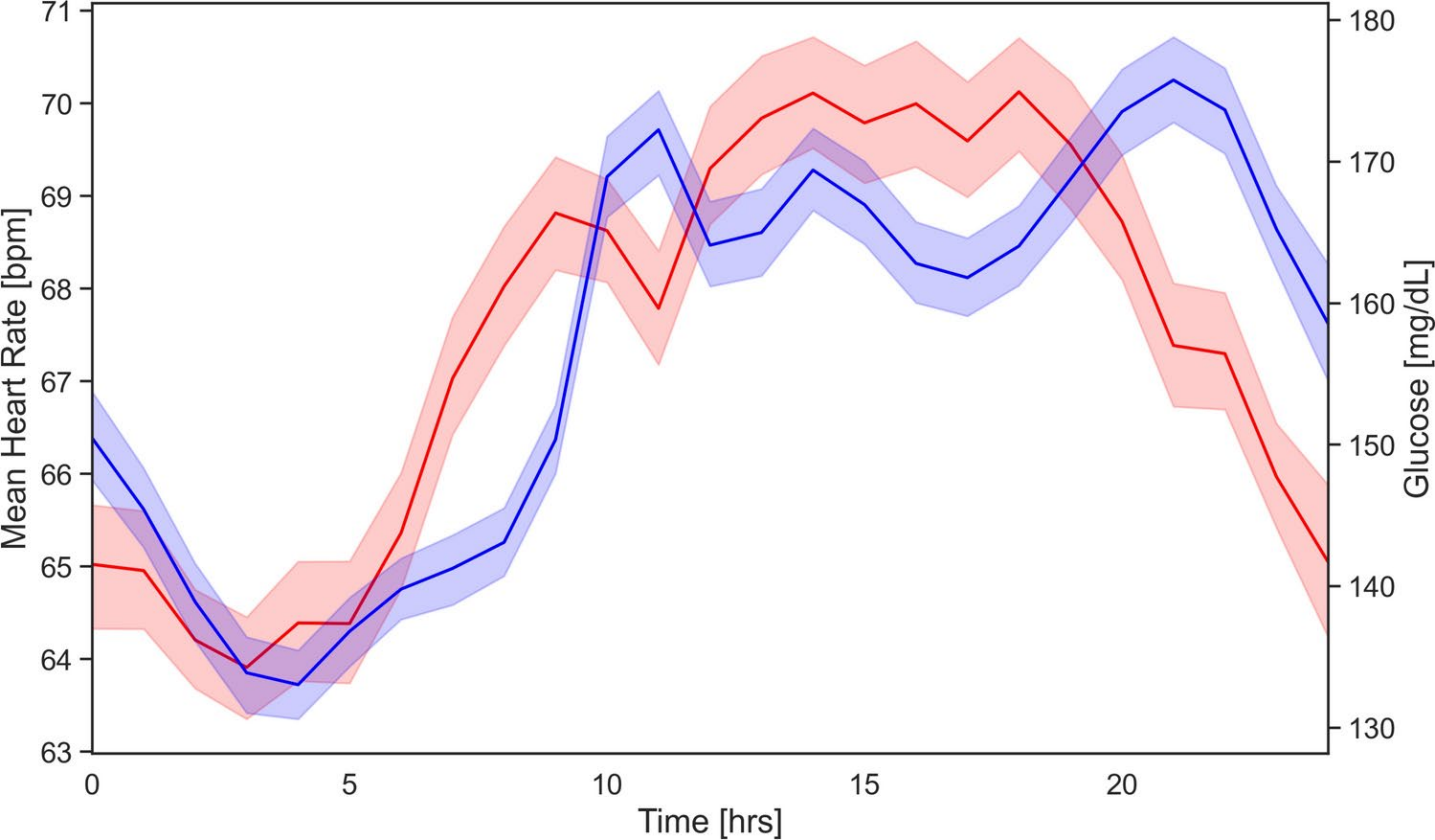
Association of glucose individualizes relative glucose levels and heart rate. The red line represents the mean heart rate of all patients over a full day, with a 95% confidence interval. The blue line shows the mean glucose levels for all patients during the same period, also with a 95% confidence interval. The correlation between glucose and heart rate is strong, with a coefficient of 0.704 and a highly significant *P* value of $8.48 \times 10^{-5}$.

Next, we investigated the association between glucose levels and the time of occurrence of bradycardia events. Figure 4 illustrates the mean heart rate and glucose levels for all patients over a full day, along with a 95% confidence interval. The correlation between glucose and heart rate is strong, with a correlation coefficient of 0.704 and a *P* value of $8.48 \times 10^{-5}$. This signifies a highly statistically significant correlation, as the *P* value is well below the common threshold of 0.05. Such a low *P* value provides strong evidence against the null hypothesis, suggesting that the observed correlation is unlikely to be due to random chance.

**Fig. 5:**
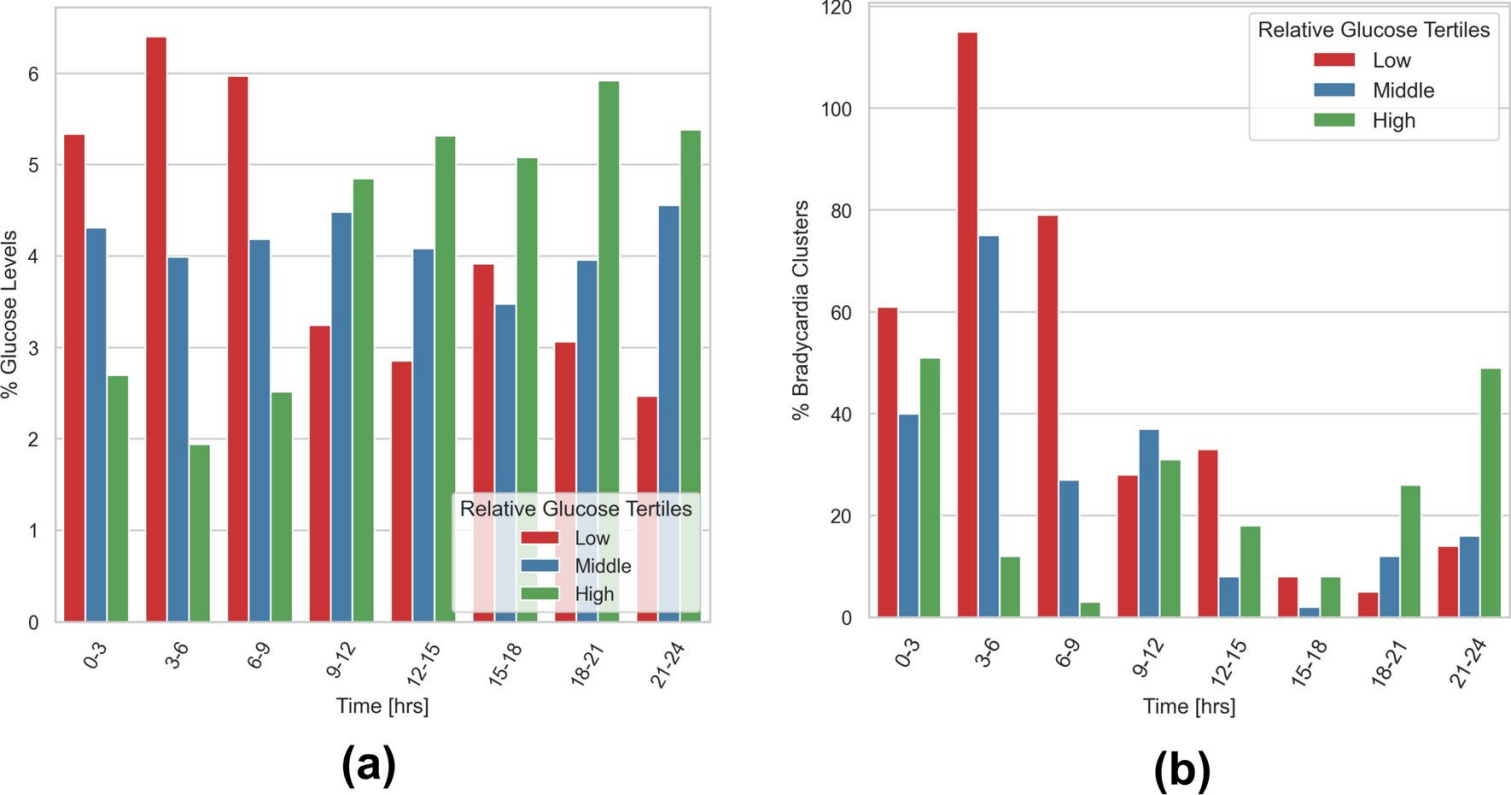
Tertiles data points percentage and three hours bradycardia percentage. (**a**) Percentage of data points at different glucose tertiles in 3-hour time intervals. **(b)** Percentage of bradycardia events at different glucose tertiles in 3-hour time intervals.

In Figure 1b, we demonstrated that the occurrence of bradycardia events is dependent on time. A similar trend is observed in the shifts of relative glucose tertile distributions across 24 hours. Notably, there is an enrichment in the high glucose tertile before midnight, followed by an overexpression of the low glucose tertile after midnight (Figure 5a). Combining all three factors (time, relative glucose tertiles, and bradycardias frequency), we observed that most bradycardias occur after midnight (from 12:00 AM to 09:00 AM) at glucose levels in the low individual tertile (Figure 5b). In contrast, bradycardias are enriched in the highest glucose levels in the evening (from 6:00 PM to 12:00 AM).

**Fig. 6:**
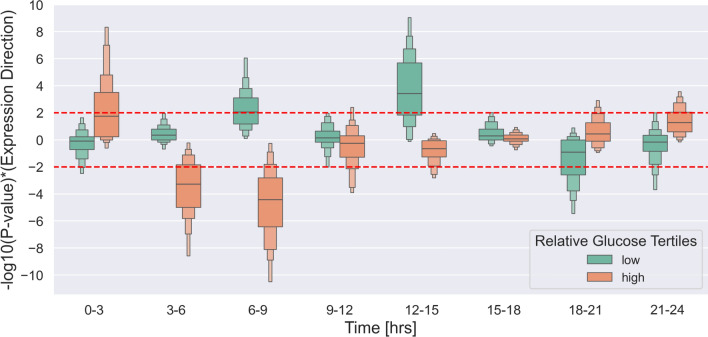
The ratio of bradycardia episodes in relation to low and high relative glucose tertiles. The figure shows the distribution of *P* values multiplied by the expression direction of bradycardia events at low and high relative glucose tertiles in 3-hour intervals, obtained using Fisher’s exact test in a bootstrap sampling strategy. Positive values indicate an increase in bradycardia events, while negative values indicate a decrease.

To quantify this difference, we conducted a Fisher’s exact test to study whether the ratio of bradycardias in each glucose tertile is identical to the ratio of overall time intervals in the corresponding tertile at each 3-hour time period (Null hypothesis) or not (Alternative hypothesis). The statistical test was performed in a bootstrap sampling fashion with 100 times iteration, and the *P* value multiplied by the expression direction was computed in each iteration. The resulting positive values for $-\log _{10}(\textit{P} \; value) \times (Expression \; Direction)$ indicate an increase in bradycardia events, while negative values represent a decrease. Figure 6 depicts the obtained result for low and high relative glucose tertiles at each time interval showing an enrichment of Bradycardia events at low relative glucose levels between 06:00 and 09:00, as well as 12:00 and 15:00, which indicated a higher risk of bradycardias event at these time intervals during a drop in glucose level. At other time intervals, we do not see any significant effect of glucose levels on the bradyarrhythmia occurrence. On the other hand, there is a high risk of such episodes between 21:00 and 03:00 during high glucose levels. However, this pattern alters quickly after 3:00, and the risk remains mostly at low glucose levels.

### Association between HRV features and bradycardia events

**Fig. 7:**
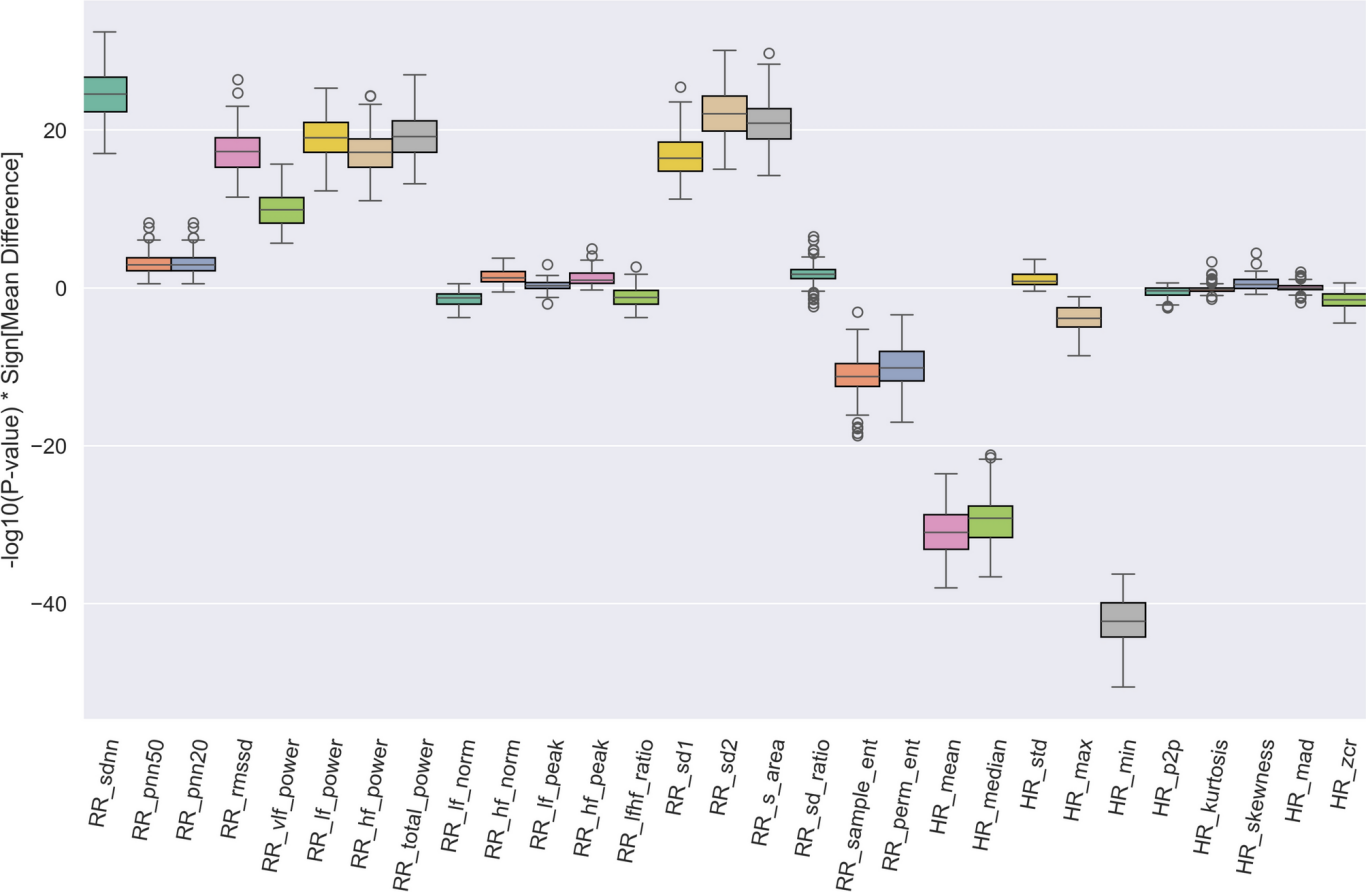
HRV features significance test in differentiating bradycardia events. A statistical significance test was performed to explore whether HRV feature values were able to differentiate the data points with bradycardia events from time points without bradyarrhythmia. In a bootstrapping approach, the distribution of *P* values was computed and multiplied by the difference between the two groups’ mean values for each HRV feature.

The association between HRV features and the bradycardia events is another question we addressed. To estimate whether the HRV changes in the course of bradycardia episodes, we performed a Wilcoxon Ransum significance test between the distribution of each HRV parameter at time windows containing bradycardia episodes and windows without such events. Nevertheless, the size of windows with bradyarrhythmias is much smaller than those in the absence of bradycardias (827 to 35415). Hence, to reduce the effect of disproportions distributions in the results, we used a bootstrap sampling technique in a loop with 100 times repetition, where small samples with a similar size (200 sample points) from each class were selected, and corresponding *P* values were computed. Figure 7 shows the distribution of the calculated *P* value of each feature multiplied by the expression direction (the sign of the difference between two groups’ means value).

The result shows a statistically significant increase in RR-sdnn, RR-rmssd, Low- and High-frequency power, as well as Poincaré plot parameters (RR-s-area, RR-sd1, RR-sd2) during Bradycardia episodes. The elevation of Poincaré features is expected due to their correlation to RR interval short- and long-term volatility (RR-sdnn and spectral powers). On the other hand, as anticipated, a pronounced drop in heart rate mean, median, and minimum values can be noticed. Further, we can see a slight decrease in sample and permutation entropies and heart rate max value, together with an elevation in VLF power in the course of bradyarrhythmias.

### Forecasting the bradycardia events

**Fig. 8:**
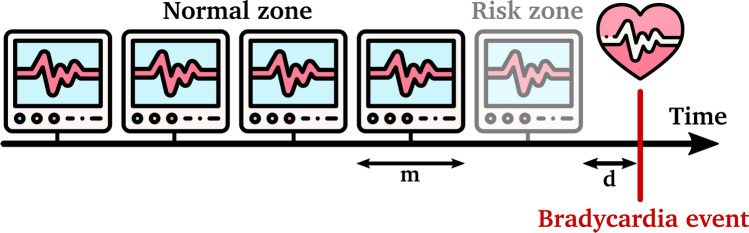
Bradycardia risk zone and normal zone schema. Bradycardia risk zone and normal zone identification: Black windows, with the length m, are the normal data points, and the gray one is the risk data point that occurred d minutes before the bradycardia event.

Considering the findings in the sections above and the relationship between the extracted HRV features, glucose value, and daytime, we explored whether these parameters can be used for forecasting bradycardia events in diabetic patients. For this purpose, we created a classification model where each feature window was considered a data point. To build the classifier, we identified and labeled all windows 5 minutes prior to the bradycardias as risk zones and labeled all the windows without and far from such events as normal zones (Figure 8). We removed all windows containing at least one bradycardia event, since predicting such windows as the target class does not have a forecasting advantage. In short, the model aims to forecast the bradyarrhythmias by classifying the risk zones from the normal zones.

Before proceeding to the classification part, we normalized each patient’s data points using the Z score and then pooled all the patients’ data. Due to the rare occurrence of arrhythmia episodes in these patients, the labeling approach leads to imbalanced classes in the extracted feature data (28873 normal points and 546 risk points in all the patients). In order to handle the imbalanced classes in the data, a bootstrap technique was employed, where at each run, we took a sample without replacement with a similar size of risk class from the normal group. Afterward, we applied a Random Forest classifier using 5-fold stratified cross-validation on the merged sampled normal and fixed risk groups. In each fold, 80% of the data (four folds) was used as the training set, and 20% (one fold) was used as the test set to evaluate performance. This process was repeated across 50 independent iterations of cross-validation, and for each iteration, the mean prediction score was recorded. Finally, the overall performance was summarized by reporting the average and standard deviation of the prediction scores across all 50 iterations.

As mentioned in the Material and Methods section, bradycardia arrhythmia is annotated in the data based on an absolute definition (hr < 45 bpm). Hence, we assumed that scaling data might affect the classifier’s performance, and since the Random Forests classifier does not require normalized data, we utilized this algorithm. After tuning and finding the optimal parameters and running the described process using each classifier, we discovered that the Random Forests model showed the mean AUC = 0.94.

**Fig. 9:**
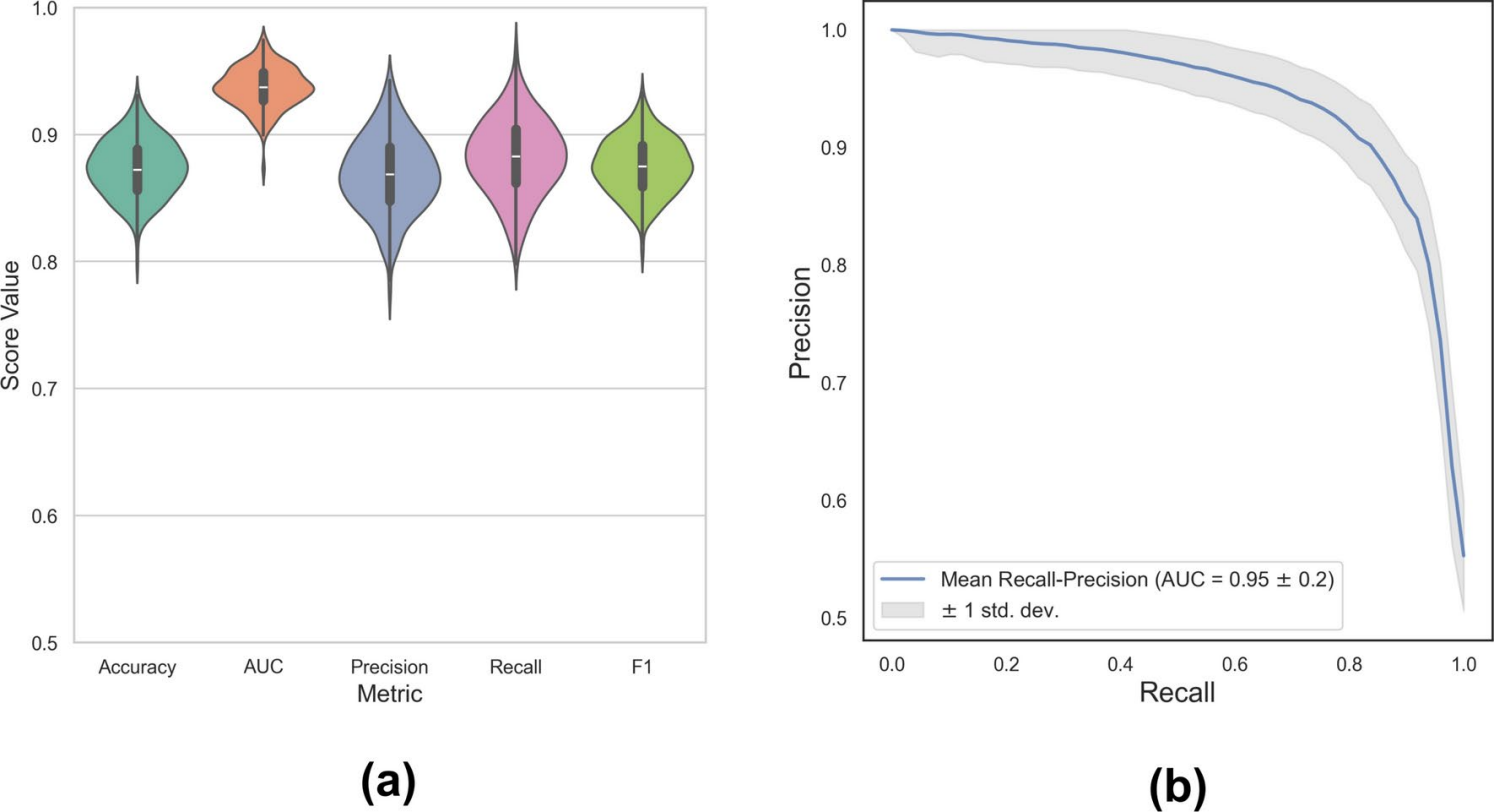
Classification measures. (**a**) The distribution of classification measures of bradycardia risk prediction using Random Forests. **(b)** The recall-precision curve of Random Forests classifier trained on the HRV features.

The distribution of the classification metrics obtained using Random Forests from bootstrap sampling, including accuracy, AUC, precision, recall, and F1 score, are displayed in Figure 9a demonstrating that all measures show an acceptable to the perfect performance of the model in identifying risk zones.

For a practical alarm system in a clinical setting, the essential factors are the model’s sensitivity and recall, where predicting the maximum number of events is of interest as well as minimizing the occurrence of false alarms. Although our model shows good accuracy and acceptable recall, the precision might not satisfy the second condition since, for such an imbalanced dataset, any precision value below 1 might not be applicable for a warning system as it leads to several false alarms. Figure 9b displays the recall-precision curve with a mean AUC of 0.94. In the data preparation step, we normalized patients’ data individually as a required step before classification.

**Fig. 10:**
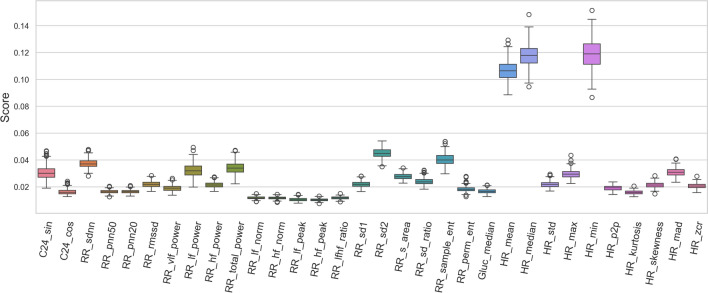
Feature Importance. A Random Forests classifier was utilized to predict the bradycardia risk in diabetic patients in a bootstrapping manner. The model returns a score showing the percentage of each feature’s involvement in the prediction. Accordingly, the distribution of each feature’s score was obtained.

Finally, we explored the significance of each feature in separating the risk zones from the normal zones using the Random Forest classifier. Figure 10 reveals the distribution of each feature’s importance scores, where we see that heart rate lowest, median, and mean values are the most critical parameters in classifying the risk windows.

## Discussion

In our present investigation, we delved into the correlation between glucose levels and heart rate parameters, examining their association with bradycardia events in 19 patients diagnosed with both diabetes and CKD who were undergoing insulin treatment. While prior studies^8,21^ have hinted at a link between bradycardia episodes and absolute hypoglycemia levels (defined as glucose values below 70 mg/dL), our analysis revealed that only a minimal fraction of such events (4%) transpired at glucose levels of 70 mg/dL or lower, whereas a relatively higher proportion (28%) manifested at glucose levels surpassing 180 mg/dL. Given the marked interindividual glucose variability within our patient cohort, we posited that a relative hypoglycemia definition might offer a more suitable framework for elucidating the connection between extreme glucose levels and bradyarrhythmias. Consequently, we stratified the glucose levels of each patient into three tertiles and observed that over 45% of bradycardias occurred in the lowest tertile of individual glucose levels.

Additionally, we conducted a statistical bootstrap analysis, affirming that bradycardia events occurred more frequently at the newly defined low glucose levels. Moreover, we investigated the influence of time on the occurrence of these events employing analogous statistical methodologies. Our findings revealed a heightened incidence of bradycardia episodes post-midnight and in the morning, aligning with a decline in relative glucose levels. Notably, these events were significantly concentrated between 06:00 and 09:00, particularly occurring at relatively low glucose levels.

Hypoglycemia episodes, especially at night, have been shown to be associated with an elevated risk of bradycardia in patients with diabetes^21^. Bradycardia itself is one of the arrhythmias contributing to the “dead in bed” syndrome. Current thinking suggests that during hypoglycemia the initial sympathetic response to hypoglycemia is followed by a parasympathetic (vagal) response. Still, under certain conditions, e.g. at night, the nocturnal sympathoadrenal response seems to be blunted thus leading to a relatively increased parasympathetic activity which then decreases the heart rate^8^.

Furthermore, we noted an increase in bradycardia episodes at relatively low glucose levels between 12:00 and 15:00, despite a general uptick in glucose levels during this timeframe. Conversely, bradycardia episodes occurring between 21:00 and 03:00 exhibited a stronger association with relatively high glucose levels. To our knowledge, our findings indicate, for the first time, that the threshold for bradycardias associated with hypo- and hyperglycemia is related to individual relative glucose levels.

In investigating the association between bradycardia frequency and glucose levels, we employed a within-patient analysis, focusing on the patients who experienced bradycardia. By analyzing bradycardia episodes across glucose tertiles within each patient, we controlled for interindividual differences, as each patient served as their own control. This approach isolated the effect of glucose levels on bradycardia occurrence, independent of confounders such as age, BMI, and cardiovascular conditions.

We also examined the expression of time-domain, frequency-domain, and nonlinear HRV features during the bradycardia events. Conducting a statistical bootstrap test, we found the overexpression of RR intervals standard deviation and spectral power as well as Poincaré plot parameters. A high Poincare plot parameter may indicate an increased risk of cardiovascular disease, as it suggests a reduced ability of the body to maintain homeostasis and respond to stressors^29^. We also observed a statistically significant drop in the mean, median and minimum heart rate values, which was a trivial outcome. Such a decline in entropy parameters was observed too.

Subsequently, we evaluated the predictive capacity of glucose values, daytime features, and HRV features in anticipating bradyarrhythmias by employing a classification framework that distinguished data points close to these events (risk zones) from others (normal zones). We utilized a Random Forests classifier for classifying the risk data points, yielding a commendable AUC of 0.94.

The analysis of parameter importance in classification has shown that heart rate mean and minimum values, as well as RR intervals standard deviation, length of an ellipse fitted on Poincaré plot (RR-sd2), sample entropy, and frequency power, play a significant role in identifying the onset of arrhythmia episodes. Variance and autocorrelation are believed to support detecting critical transitions in dynamic systems^30^. Our findings support this assumption, as standard deviation is derived from variance, and RR-sd2 represents the autocorrelation coefficient.

This study has several limitations that warrant further investigation in future research endeavors. First, while the predictive model demonstrates respectable precision (0.87) and sensitivity (0.88) in forecasting bradyarrhythmias, the imbalance in the dataset-with fewer risk zones than normal zones-hampers the practical utility of the model. This is because enhancing precision to minimize false alarms inevitably diminishes sensitivity. Second, the constrained availability of data samples impedes the development of a generalizable machine learning model for predicting bradyarrhythmias. Third, the small sample size of 19 patients introduces a risk of bias, as it may not adequately capture the diversity of glucose-bradycardia relationships across a broader population of patients with diabetes and CKD. In addition, our study did not focus on clinical events and only evaluated the association of changes in glucose levels with bradycardia. Finally, we did not explore the effects on other arrhythmias such as premature ventricular beats or ventricular tachycardias.

Our results underscore the significance of implementing a personalized glucose monitoring approach for individuals with diabetes and CKD. Customizing monitoring and care to suit the distinct requirements of these high-risk patients enables healthcare providers to deliver more targeted and efficient patient-centric interventions. This personalized strategy has the potential to enhance the prevention and management of health complications within this vulnerable demographic.

## Supplementary Information

Supplementary Information.

## Data Availability

The data included in this study, contain sensitive health-related information. Due to the small data set, anonymization techniques, like e.g. k-anonymity, cannot be applied usefully without a relevant loss of information. Thus, according to the Health Data Protection Act North Rhine-Westphalia (Gesundheitsdatenschutzgesetz NRW) and the internal guidelines of the Data Protection Officer of the University Hospital RWTH Aachen, the raw patient data must not be made publicly available, since total anonymization cannot be guaranteed. However, researchers who are interested in the data, may the corresponding author with a statement about which research questions they aim at and which data are necessary for this purpose. Then, in a bilateral process, a solution for the data exchange can be found in compliance with legal and ethical restrictions
