# Supplementary material for: Monitoring individualized glucose levels predicts risk for bradycardia in type 2 diabetes patients with chronic kidney disease: a pilot study
Source: Sci Rep. 2024 Dec 5;14:30290. doi: 10.1038/s41598-024-81983-x (PMC11621348; doi:10.1038/s41598-024-81983-x)
Supplement: Supplementary file 1 — Supplementary Information. [file 41598_2024_81983_MOESM1_ESM.pdf]

Supplementary Material

Supplementary Methods

Data preprocessing and feature extraction: Denoising. We started with cleaning and removing the outliers in the RR intervals measurement in the windowing process. First, we removed the RR intervals out of the physiological accepted range (200 ms - 2500 ms). Next, we removed the ectopic or artifact RR intervals using a recursive filtering procedure [1]. Ectopic beats refer to premature ventricular and atrial contraction. Although ectopic beats are considered normal and do not indicate cardiac pathology, they can affect the analysis of the HRV and other types of arrhythmias.

The glucose values outliers ($< 10\frac{mg}{dL}$ and $> 700\frac{mg}{dL}$) were also removed during the windowing procedure.

Data preprocessing and feature extraction: Missing value imputation. Missing value imputation is another crucial step before performing HRV analysis. It is shown that missing values can cause misleading results in HRV estimation [2]. The missing values are present in the data either due to monitoring error (detachment of the sensors) or after removing the outliers. We implemented a linear interpolation to handle the missing RR intervals, where the number of missing values between two available values is less than 5. We dropped the window in the presence of long missing values as it can immensely affect the results.

Supplementary Results

We executed a Fisher's Exact test in a bootstrap sampling manner to examine whether the unequal occurrence of bradycardia events in the relative glucose tertiles is statistically significant. The Null hypothesis is that the ratio of bradycardia number in each tertile to the whole number of events is similar to the glucose levels ratio in each tertile (1/3), while the alternative hypothesis is that the bradycardia frequency ratio is significantly different from the glucose levels ratio.

We selected a sample of patients with replacement iteratively in this approach (100 times) and computed the P value for each tertile multiplied by the expression direction. The expression direction can be calculated as the sign of the difference between the ratio of bradycardia events in each tertile and the glucose ratio in the corresponding tertile. A positive value indicates the over-expression of bradycardia events in the tertile, while the negative value shows the under-expression of bradycardia occurrence.

As anticipated, bradycardia events were significantly pronounced in the low relative glucose tertile while we observed, in most iterations, a low happening of bradycardias in normal and high glucose tertiles (Supplementary Figure S1).

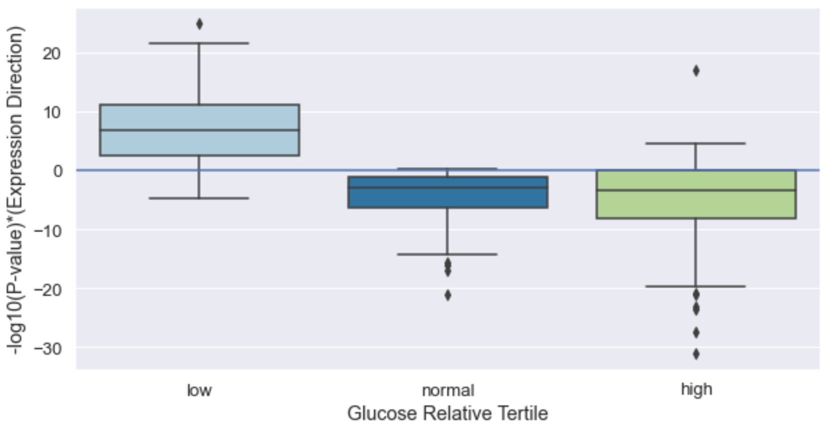

Supplementary Figure S1. Bradycardia episodes in relation to relative gluocse tertiles. The distribution of tertiles P values multiplied by the expression direction of bradycardia events (enriched or not enriched) in each relative glucose tertile obtained by Fisher's Exact test in a bootstrap sampling strategy.

References

[1] M. Karlsson, R. Hörnsten, A. Rydberg, and U. Wiklund, “Automatic filtering of outliers in rr intervals before analysis of heart rate variability in holter recordings: a comparison with carefully edited data,” *Biomedical engineering online*, vol. 11, pp. 1–12, 2012.

[2] K. K. Kim, J. S. Kim, Y. G. Lim, and K. S. Park, “The effect of missing rr-interval data on heart rate variability analysis in the frequency domain,” *Physiological measurement*, vol. 30, no. 10, p. 1039, 2009.
